# Nutrition Patterns, Metabolic and Psychological State Among High-Weight Young Adults: A Network Approach

**DOI:** 10.3390/nu18010145

**Published:** 2026-01-01

**Authors:** Geovanny Genaro Reivan Ortiz, Roser Granero, Laura Maraver-Capdevila, Alejandra Aguirre-Quejada

**Affiliations:** 1 Faculty of Clinical Psychology, Catholic University of Cuenca, Cuenca 010107, Ecuador; greivano@ucacue.edu.ec; 2 Department of Psychobiology and Methodology, Universitat Autònoma de Barcelona, 08193 Barcelona, Spain; laura.maraver@uab.cat; 3 Ciber Fisiopatología Obesidad y Nutrición (CIBERobn), Instituto Salud Carlos III, 28029 Madrid, Spain; 4 Group of Psychoneurobiology of Eating and Addictive Behaviours, Neuroscience Program, Bellvitge Biomedical Research Institute (IDIBELL), 08908 Barcelona, Spain; 5 Nursing Career, Catholic University of Cuenca, Cuenca 010107, Ecuador; maaguirreq@ucacue.edu.ec

**Keywords:** nutritional patterns, cardiometabolic risk, HOMA-IR, obesity

## Abstract

Background and Objectives: Studies suggest that overweight and obesity are major risk factors for various metabolic and psychological disorders, and that a better understanding of the interactions between these factors may lead to more effective intervention strategies. The main aim of this study is to examine the structure of interrelationships among sociodemographic characteristics, nutritional patterns (NP), metabolic indicators, and psychopathological measures using network analysis in a sample of young university students with overweight and obesity, and to identify the most central variables and their empirical groupings. Methods: N = 188 overweight/obese young adults participated, university students, men and women, aged 18 to 25 years. Results: The variable with the highest centrality (relevance and connectivity capacity) was stress level, identified as the bridge node. Two other important features were an NP characterized by vitamin and mineral consumption, and the presence of arterial hypertension (HTN). Three clusters of nodes emerged, grouping: (a) insulin, glucose and Homeostatic Model Assessment for Insulin Resistance (HOMA-IR index); (b) cholesterol and triacylglycerol; and (c) sociodemographic profile, psychological state, BMI and HTN. Conclusions: The results highlight stress levels as a central factor influencing the metabolic and mental health of overweight/obese young adults. Interventions aimed at reducing stress and improving nutrition patterns are crucial for improving the overall wellbeing of these individuals.

## 1. Introduction

Overweight and obesity are considered public health problems due to their high prevalence and severe implications for both individual health and national healthcare systems [1]. These conditions not only affect people’s quality of life, but also significantly increase the risk of developing chronic diseases, such as type 2 diabetes, cardiovascular disease, arterial hypertension (HTN), certain types of cancer, and respiratory and musculoskeletal disorders [2]. In addition, the costs associated with the treatment of these diseases are continuing to rise, generating an economic burden for both public and private health systems [3].

Although overweight and obesity pose risks at all ages, young adults face particular challenges that can make the consequences of these conditions even more severe than they are for children, adolescents, and older adults [4]. In addition to the physical effects, there are psychological and social repercussions that can impair their emotional well-being, academic performance and social integration [5]. Furthermore, obesity at this stage of life can have long-term consequences, as unhealthy eating habits formed during university years are more likely to persist into adulthood [6].

Indeed, research indicates that overweight or obese university students tend to have unhealthy, high-calorie eating habits, due to the excessive consumption of sugars, saturated fats, and sodium, such as those found in fast food, soft drinks, and ultra-processed products [7,8,9]. Food that is rich in refined carbohydrates and saturated and industrial trans fats promotes weight gain and increases the risk of metabolic and cardiovascular diseases. In addition, a lack of essential nutrients (such as lean proteins, fiber, vitamins, and minerals) can lead to deficiencies that further affect health. Inadequate portion control and irregular meal times are other factors that contribute to an unhealthy lifestyle pattern that makes it difficult to control body weight [10,11,12]. Sleep-related behaviors frequently marked by shortened sleep periods and suboptimal sleep quality, conditions reported by 10% to 50% of university students [13], represent another key component of unhealthy lifestyle patterns in this group. Current evidence shows that inadequate sleep routines, including late-night internet use, in combination with nighttime eating and other nocturnal activities, are associated with increased risk of being overweight and additional adverse health indicators [14]. Furthermore, research consistently points to a U-shaped association between sleep duration and self-perceived health, whereby both insufficient and excessive sleep are linked to poorer health outcomes [15].

Obesity can be classified into metabolically healthy obesity (MHO) and metabolically unhealthy obesity (MUO) [16,17,18], a clinically relevant distinction that captures heterogeneity in metabolic functioning among individuals with obesity. While both groups may present similar levels of excess body weight, they differ in metabolic profiles and cardiometabolic risk, which has important implications for clinical assessment and intervention. While MHO is characterized by the absence of major metabolic alterations, MUO is associated with insulin resistance, dyslipidemia, hypertension, and an increased risk of type 2 diabetes and cardiovascular disease [17,18,19,20]. Emerging evidence also links MUO to elevated liver enzymes (e.g., ALT, AST, and GGT) [21,22], which are associated with hepatic insulin resistance and clustered cardiometabolic disturbances, independently of overt liver disease or alcohol consumption [23]. But despite the classification into MHO versus MUO, it should be noted that MHO represents only a milder cardiovascular risk profile, and maintaining a normal weight remains the most beneficial condition for overall health, as both overweight and obesity carry significant long-term health risks.

Metabolic health status is commonly defined using cardiometabolic risk criteria. One method is based on the criteria modified by the International Diabetes Federation [24], which defines MUO as the presence of at least two of the following cardiometabolic risk factors: elevated triglycerides (≥150 mg/dL), high fasting glucose (≥126 mg/dL), reduced HDL-cholesterol (<45 mg/dL), and high blood pressure (≥120/80 mmHg) (this method classifies individuals with one or none of these factors as MHO). The second method attributes MUO to individuals with a HOMA-IR score ≥ 3 [25,26,27]. Although both approaches are widely used, there is currently no consensus regarding the optimal method for young adults. The HOMA-IR–based classification is commonly employed in clinical research, including studies with young adult populations, as it directly reflects insulin resistance, a central feature of metabolically unhealthy obesity. University students, due to poor eating habits, stress, sedentary lifestyles and unbalanced diets, are particularly prone to developing MUO, creating a vicious cycle that aggravates both their physical and mental health [28,29,30].

### 1.1. Factors Associated with Overweight and Obesity Among Young Adults

Nutritional patterns (NP) play a key role in the relationship between overweight and obesity [31,32,33]. Among university students, multiple factors, such as academic stress, lifestyle changes, time constraints, and limited access to healthy foods, can negatively influence eating habits. Deficiencies in essential nutrients (e.g., vitamin D, calcium, and magnesium) can also increase hunger and reduce physical activity, contributing to weight gain [31,32,33,34]. Excessive consumption of simple carbohydrates, such as refined sugars and processed foods, generates insulin spikes that promote fat storage, while saturated and trans fats (present in fried and processed foods) are other contributors [9]. Furthermore, excess sodium, which is common in fast food, can lead to fluid retention and is associated with the over-consumption of ultra-processed foods [35,36].

Studies also find that sociodemographic variables, such as sex, marital status, socioeconomic status, and age, influence the risk of overweight and obesity even among young adults [37,38,39,40]. For example, men tend to accumulate more abdominal fat, while women face additional risks due to hormonal changes [41]. Single or divorced individuals often adopt less healthy eating habits, while married couples may gain weight due to shared eating behaviors [42,43]. People of lower socioeconomic status face barriers to accessing healthy foods and exercise [44], while older adults experience a slowdown in metabolism and loss of muscle mass, which increases the risk of being overweight [44,45,46].

Beyond environmental influences, an expanding body of evidence has examined the role of female reproductive factors in the development of cardiovascular conditions [47]. These studies highlights that certain reproductive periods or events (such as adverse pregnancy outcomes, endocrine-related reproductive disorders, recurrent preeclampsia, and the first decade postpartum among women with a history of gestational diabetes) are associated with substantially elevated risks for coronary heart disease. In parallel, lifestyle behaviors, particularly habitual levels of physical activity, have been consistently identified as key determinants of cardiometabolic health, with insufficient activity linked to adverse outcomes including elevated blood pressure, dyslipidemia, increased body mass index, and higher incidence of cardiovascular disease [48]. Low levels of physical activity are also associated with sarcopenic obesity (the concurrent presence of reduced muscle mass and strength alongside excess adiposity) which is increasingly recognized as a condition that exacerbates health risks beyond those of obesity alone (even among young individuals), including elevated cardiometabolic risk and greater likelihood of disability [49,50].

Furthermore, emotional factors such as anxiety, stress, and depression play a crucial role in overweight and obesity among university students [51,52,53,54]. These difficulties can induce “emotional eating,” whereby students turn to high-calorie foods to alleviate their emotional distress [53]. Stress raises cortisol levels, which increases cravings for unhealthy foods, while depression reduces the motivation to observe healthy habits [52]. The interaction between these factors creates a vicious cycle of poor eating habits and a sedentary lifestyle, thus further increasing the risk of obesity and the negative impacts on both physical and mental health [51,54].

### 1.2. Justification and Aims of the Study

Given the interrelated nature of nutritional patterns, metabolic status, and psychological factors in overweight and obesity among young adults, a network approach provides an appropriate framework to capture the complexity of these interactions in young adults [55,56]. Such an approach enables the identification of key variables, their relative importance, and their organization into interconnected clusters, thereby informing more comprehensive and personalized intervention strategies.

Accordingly, this study applies network analysis to examine the relationships among physical, psychological, sociodemographic, and clinical correlates of overweight and obesity, to identify the most central nodes within the network, and to detect empirically derived clusters of interrelated variables. The analyses were conducted on data from young university students (aged 18–25 years) characterized by overweight or obesity, as they represent a high-risk population. These subjects exhibit nutritional patterns that are generally unhealthy and are associated with multiple physical and mental health problems. If early interventions are not implemented, these nutritional patterns may persist over time, potentially exacerbating overall health outcomes.

## 2. Materials and Methods

### 2.1. Participants

This study analyzed data collected as part of a broader research project aimed at identifying factors influencing the cardiometabolic health of young university students with overweight or obesity. A comprehensive description of the sampling procedures, recruitment strategies, and participant characteristics is provided in the study by Reivan and colleagues, which readers can consult for full methodological details [57]. The sample included N = 188 overweight and obese students recruited from the Catholic University of Cuenca, in Azogues, Ecuador. All participants were volunteers and provided informed consent. Exclusion criteria included pregnancy, endocrine or genetic disorders (such as hypothyroidism, type 1 diabetes, and Cushing’s syndrome), a weight-loss diet, or the use of supplements or medications that could affect blood glucose, lipid profile, body weight, or blood pressure. Candidates with eating disorders were not excluded, since the aim of the study was to obtain evidence from a general population of overweight or obese young adults, both with and without eating disorders, thus enhancing external validity and facilitating generalization of the findings to a broader and more representative population.

### 2.2. Measures

*Nutritional patterns.* The Food Frequency Questionnaire (FFQ) [58] was used to assess the participants’ diet with the assistance of a trained nutritionist, who asked them to note how frequently they consumed certain foods (daily, weekly, or monthly) and the amount ingested in the past 12 months, based on standard portions. Total energy and nutrient intake were calculated by summing the values of all foods consumed. Nutrimind software (https://www.nutrimind.net/) was used to manage this data.

This study analyzed the scores from three nutritional patterns (NP), the full composition and variables of which are defined in Reivan-Ortiz et al. [57]. These nutritional patterns were derived through factor analysis, meaning that they do not represent direct measurements of specific components, but rather reflect the grouping of all components provided by the FFQ scale into coherent factors (which proved good reliability and validity indexes). NP1 (high mineral and vitamin content) is characterized by a high intake of potassium, magnesium, folate, pantothenic acid, riboflavin, phosphorus, zinc, calcium, vitamins B12, B6 and C, and fiber; NP2 (high carbohydrate) is characterized by a high intake of thiamine, niacin, carbohydrates and iron; and NP3 (high fat and sodium) is characterized by a high intake of polyunsaturated fatty acids, sodium, saturated fatty acids and monounsaturated fatty acids.

*Cardiometabolic Health Factors.* A specialized nurse with extensive experience in nutrition assessed anthropometric indices and cardiometabolic risk factors. Weight was measured using a calibrated electronic scale (BCS-G6) with an accuracy of 0.1 kg. Height was recorded with a stadiometer accurate to 0.1 cm. Waist circumference (WC) was measured twice for each participant, and the average of both measurements was considered the definitive value [59]. After a 5 min rest, diastolic blood pressure (DBP) and systolic blood pressure (SBP) were recorded twice at a 15 min interval on the right arm [60], with the average of the two measurements taken as the final value. Biochemical values were obtained from venous blood samples taken in a seated position after a 12 h fast. Blood glucose levels, lipid profiles, and insulin concentrations were also measured. Insulin resistance was calculated using the Homeostasis Model Assessment of Insulin Resistance (HOMA-IR = [insulin (µIU/mL) × glucose (mg/dL)]/405) [61].

*Anthropometry.* Body mass index (BMI) was used as the primary measure of nutritional status, calculated by dividing weight in kilograms by height in meters squared. In line with the World Health Organization [62], BMI was classified into two categories: overweight (25 < BMI < 30) and obesity (BMI > 30).

*Psychological status.* It was assessed with the Depression, Anxiety, and Stress Scale (DASS-21) [63], a self-administered questionnaire consisting of 21 items with responses rated on a Likert scale ranging from 0 (“never”) to 3 (“almost always”). This scale was designed to measure symptoms associated with depression, anxiety, and stress experienced in the previous week, and also provides a total score reflecting general psychological distress. In this study, internal consistency was considered good to excellent, with a Chronbach’s alpha coefficient of 0.80 for depression, 0.82 for anxiety, 0.86 for stress, and 0.90 for the total scale.

*Socioeconomic factors.* The participants’ age, gender, marital status, and socioeconomic status (SES) were also recorded, the latter using the Hollingshead Four Factor Index [64].

### 2.3. Procedure

Participants were recruited after obtaining permission from the administration of the Catholic University of Cuenca and the program directors and professors. An invitation letter was sent to the students via institutional email.

Students who agreed to participate attended two assessment sessions. The first was held in the classroom, where two clinical psychologists and a nutritionist, all trained in the use of psychometric assessment tools, asked them to complete questionnaires collecting nutritional, psychological, and sociodemographic data. This session lasted approximately 40 min.

At the end of the first session, each student was informed of the time and date of the second session. During this second session, a nurse and a clinical psychologist, both with more than 10 years of experience, recorded anthropometric and biochemical data.

The data were collected in October 2023. Participation was completely voluntary and no financial or academic incentives were offered.

### 2.4. Network Analysis

Network analysis has emerged as a prominent framework for studying psychological and behavioral phenomena by conceptualizing them as systems of interacting components rather than as manifestations of underlying latent constructs. Instead of assuming that observed variables are driven by a single common cause, this approach focuses on the pattern of direct relationships among variables, allowing researchers to examine how individual elements within a system influence one another. This framework is particularly well suited for the study of complex and multifactorial conditions, such as obesity and health-related behaviors, where biological, psychological, and behavioral factors are dynamically interconnected. Network analysis enables the simultaneous modeling of these interdependencies and facilitates the identification of variables that may play a central role in the maintenance or propagation of the overall system [65].

In the context of mental health research, network models have been widely applied to examine symptom–symptom interactions and to provide insights into the structural organization of psychopathology. These models have contributed to a more nuanced understanding of disorders as dynamic systems, highlighting the relevance of specific symptoms or behaviors that may serve as key targets for clinical assessment and intervention [66,67]. Recent methodological developments have further strengthened this approach by providing standardized procedures for network estimation, visualization, and interpretation, thereby enhancing the accuracy, stability, and robustness of network-based findings [68].

Within a network model, the fundamental components are nodes and edges. Nodes represent the individual elements of the system, such as symptoms, behaviors, metabolic indicators, or other measured variables, whereas edges represent the statistical associations between nodes after controlling for the influence of all other variables in the network. The presence and strength of an edge reflect the degree to which two nodes are directly related.

Beyond describing pairwise associations, network analysis allows for the examination of the overall structure of the system through the identification of central nodes, which are highly connected and may exert a disproportionate influence on the network. Additionally, bridge nodes can be identified as elements that connect different clusters or domains within the network, potentially facilitating interactions between distinct processes. Finally, clusters of densely interconnected nodes may reflect functionally or clinically meaningful groupings of variables. Together, these features provide a comprehensive and integrative perspective on the organization and dynamics of complex psychological and health-related phenomena.

### 2.5. Statistical Analysis

The analysis was conducted with Gephi 0.9.2 for Windows [69], open-source multiplatform software (available at http://gephi.org) that was specifically developed for network exploration and visualization and is used to display spatialization processes and to compute essential network parameters, including centrality, density, clustering and modularity.

The study analyzed a network of nodes including sociodemographic variables (sex, age, marital status and socioeconomic status), physical variables (BMI, HTN, glucose, insulin, cholesterol-T, triacylglycerol, and HOMA-index), psychological state (depression, anxiety and stress levels), and NP (NP1, NP2, NP3). The edge weights were calculated by means of a partial correlation matrix between node pairs. These coefficients [Rp] provided the strength and direction of the relationships between nodes whilst adjusting for the other variables. Data structure resulted in 17 nodes and 136 edges. However, most of the edges had low weights (partial correlations around 0, suggesting poor association between variables). To simplify the structure and obtain a more parsimonious model, following standard procedures in network analyses with a high number of edges, we only selected edges with *p* < 0.25.

Network characterization used a range of statistics that were automatically provided by Gephi. First, three measures of centrality were obtained and interpreted: eigenvector, betweenness and closeness centrality. Eigenvector centrality, the weighted sum of all the degrees (weights) of the nodes to which a given node connects, was used as the global relevance of a node in the structure. Betweenness centrality, obtained by counting the number of times each node appears on the shortest path between two other nodes, was interpreted as that node’s capacity for providing a “bridge” between different parts of the network, and is a useful measure for identifying the nodes that, if removed, would cause a network to collapse. Nodes with high betweenness centrality achieve high dependence control over the other nodes, as they play a fundamental role in connecting the network structure and transferring/connecting information). Finally, closeness centrality is calculated as the average distance from a certain vertex in the graph to each of the other vertices. Nodes with high closeness centrality exhibit high independence from potential intermediary/control nodes, since they are connected to the other nodes through a low number of edges.

This study also identified empirical clusters of nodes, also known as communities or modules [70]. Gephi uses an automatic procedure to detect groups in the dataset, which is particularly useful when such groupings are not known a priori. Node clusters group variables that are strongly interconnected to each other and are less connected to nodes outside the cluster.

Three other global network measures were obtained [71]: (a) average path length, which is the mean of the shortest path between all node pairs and measures how efficiently information is transported within the network; (b) diameter, defined as the maximum eccentricity of any vertex in the graph and which measures the greatest distance between the two furthest nodes; and (c) density, defined as the number of modeled edges divided by the total number of potential edges, thus measuring how close the network is to completion, which occurs when a graph includes all possible edges, with a density equal to 1.

## 3. Results

### 3.1. Characteristics of the Sample

The sample consisted of 188 participants, of whom 43.1% were women (*n* = 81) and 56.9% were men (*n* = 107). Most participants were single (78.7%, *n* = 148), followed by those who were married or living as a couple (18.6%, *n* = 35), and a small proportion who were divorced or separated (2.7%, *n* = 5). Regarding socioeconomic status, 37.8% (*n* = 71) reported a low social status, 33.0% (*n* = 62) a medium status, and 29.3% (*n* = 55) a high status. Participants had a mean age of 20.8 years (SD = 2.6) and an average BMI of 28.4 kg/m^2^ (SD = 2.9). Concerning clinical conditions, 25.5% (*n* = 48) reported having hypertension, whereas 74.5% (*n* = 140) did not.

Regarding psychological measures, mean scores were 98.9 (SD = 11.2) for depression, 10.4 (SD = 2.9) for anxiety, and 193.2 (SD = 52.1) for stress. Biological indicators showed mean levels of 147.80 mg/dL (SD = 52.43) for glucose, 2.54 µIU/mL (SD = 0.78) for insulin, 5.89 mmol/L (SD = 1.30) for total cholesterol, 5.21 mmol/L (SD = 1.55) for triacylglycerol (TAG), and a mean HOMA-IR index score of 10.69 (SD = 2.62). Regarding these descriptive values, it must be considered that these biochemical measurements were performed after an overnight fast. As the study sample consisted of young adults with overweight or obesity (BMI ≥ 25 kg/m^2^), mean values of these metabolic indicators—including glucose, HOMA-IR, and related cardiometabolic measures—were higher than those typically observed in young adults of normal weight, reflecting the characteristic metabolic profile associated with increased adiposity.

### 3.2. Network Visualization

**Figure 1:**
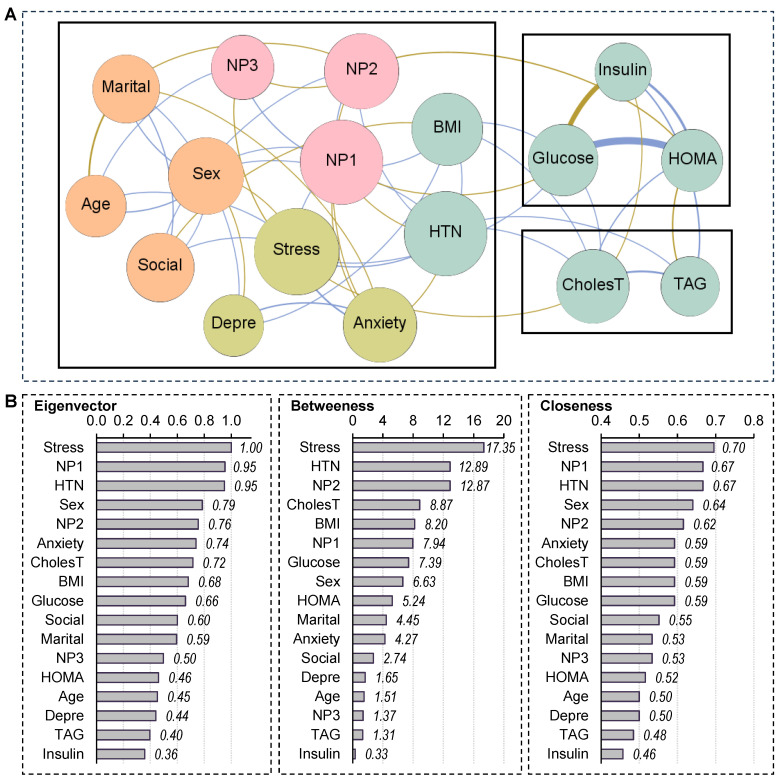
Visualization of the network. Note. (**A**): Network graph. Positive edges are represented by blue lines, and negative edges by brown-ochre lines. As thicker the edge as stronger the connection weight. Nodes are plotted in colors depending on the dimension: sociodemographic variables (orange), nutritional patterns (pink), physical variables (green), psychological variables (pistachio). Nodes are identified as: Sex (biological sex), Age (chronological age), Marital (marital status), Social (social position index), NP1 (nutritional pattern 1, minerals and vitamins), NP2 (nutritional pattern 2, carbohydrates), NP3 (nutritional pattern 3, fat and sodium), BMI (body mass index), HTN (hypertension), Glucose, Insulin, CholesT (cholesterol total), TAG (triacylglycerol), HOMA (HOMA-IR index), Depre (depression), Anxiety, Stress. Clusters of nodes are grouped inside boxes. (**B**): centrality indexes.

Figure 1 presents the visualization of the network. This figure is organized in two panels: panel A (upper part of the Figure) displays the network graph, whereas Panel B (lower panel) presents the centrality indices. Table 1 contains the complete parameters obtained in the analysis. Regarding the network graph, the nodes are color-coded by dimension: sociodemographic variables (orange), NP (pink), physical variables (green) and psychological variables (pistachio). Edges with positive weights are shown in blue to represent positive relationships and (partial correlations Rp > 0,) while negative weights are shown in ochre/brown to represent negative relationships (Rp < 0). A total of 50 edges were selected for the analysis, resulting in a network density of 0.368. The diameter was 3, and the average path length was 1.77.

Three clusters of nodes (modularity classes) were identified, and are shown inside boxes in Figure 1. One cluster grouped insulin, glucose and HOMA-IR index. Another cluster contained cholesterol and TAG, while the third cluster grouped the remaining nodes in the network. The modularity index, which describes how the network is classified into sub-networks and ranges from −1 to 1, was 0.40, suggesting fair modularity. Finally, the average clustering coefficient was 0.443.

### 3.3. Centrality Indexes in the Network

Panel B in Figure 1 displays the bar charts with the nodes ordered by their centrality coefficients. The “stress” node has the highest centrality for the three properties analyzed in the network: eigenvector, betweenness and closeness. This means that the participants’ perceived stress is the most influential variable in the graph, as it is nearest to the other variables, and is hence deemed the bridge node. The next two nodes with the highest centrality values were NP1 (eigenvector and closeness) and HTN (for betweenness).

**Figure 2:**
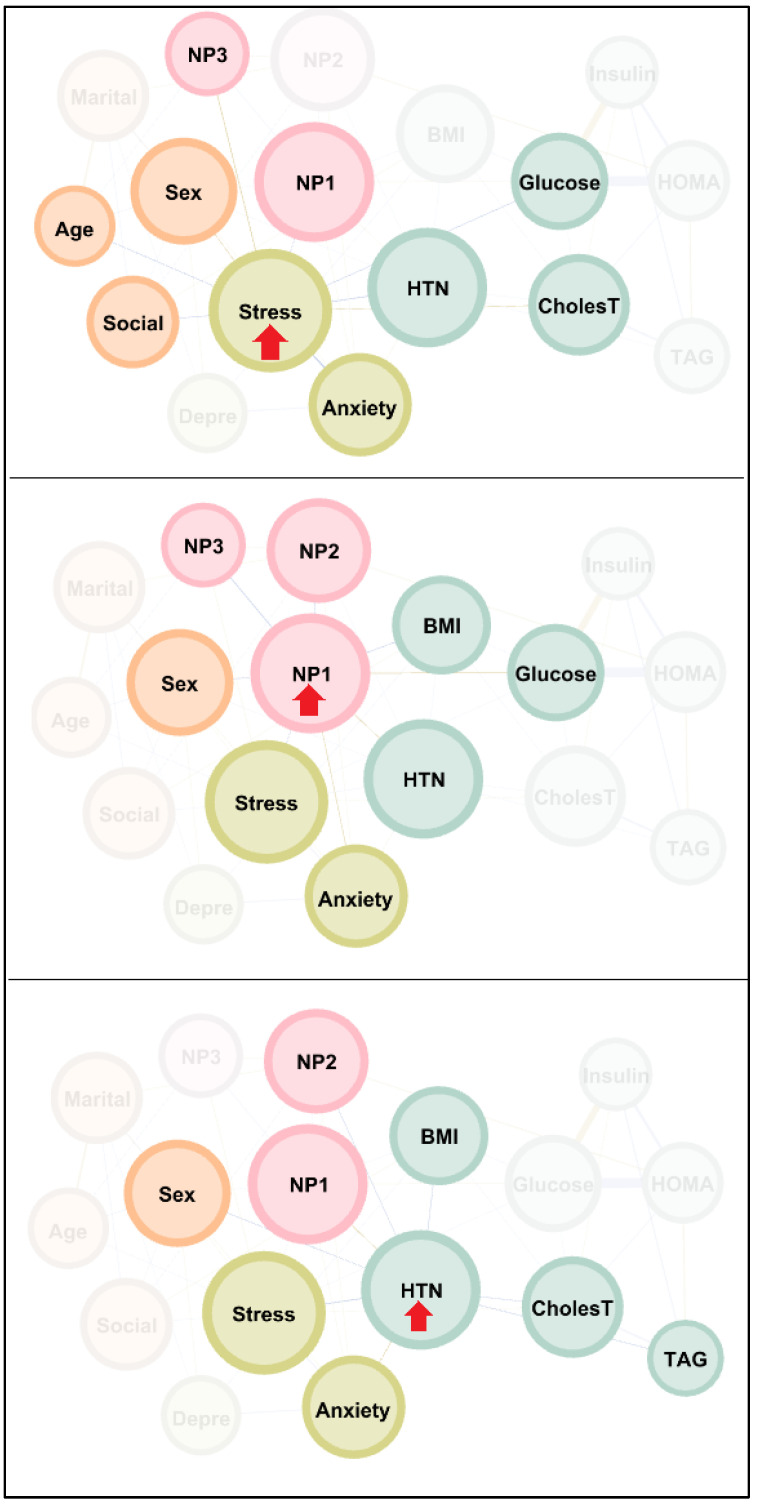
Visualization of the connections of the variables with highest centrality. Note. Nodes are plotted in colors depending on the dimension: sociodemographic variables (orange), nutritional patterns (pink), physical variables (green), psychological variables (pistachio). Nodes are identified as: Sex (biological sex), Age (chronological age), Marital (marital status), Social (social position index), NP1 (nutritional pattern 1, minerals and vitamins), NP2 (nutritional pattern 2, carbohydrates), NP3 (nutritional pattern 3, fat and sodium), BMI (body mass index), HTN (hypertension), Glucose, Insulin, CholesT (cholesterol total), TAG (triacylglycerol), HOMA (HOMA-IR index), Depre (depression), Anxiety, Stress. Clusters of nodes are grouped inside boxes.

Figure 2 shows the main connections of the nodes with the highest centrality in the network: stress, NP1 (vitamins and minerals) and HTN. Activating these nodes significantly impacted most other variables in the graph. Specifically, activation of the stress node strongly impacted anxiety, NP1 (minerals and vitamins), NP3 (fat and sodium), HTN, glucose, cholesterol, and all sociodemographic variables apart from marital status. Activation of the node containing the NP1 score (minerals and vitamins) significantly impacted the other two NPs, BMI, glucose, HTN, stress, anxiety, and sex. Activation of HTN produced an observably high impact on NP1 (minerals and vitamins), NP2 (carbohydrates), BMI, cholesterol, TAG, stress, anxiety, and sex.

## 4. Discussion

From a network approach, this study has explored the interrelations between physical and psychological health variables among overweight and obese university students. The findings provide a detailed visualization of the connections between the different nodes (sociodemographic profile, nutritional patterns (NP), metabolic status and psychological measures), and also their most important features, linkage capacity, and empirical functional modules (clusters of nodes).

In the field of nutrition, the use of network-based methodologies remains limited, as highlighted by a recent scoping review that systematically mapped how network analysis has been applied to dietary pattern research [72]. The review identified only a small number of studies using network models (most commonly Gaussian Graphical Models) to examine relationships among foods or dietary components, revealing substantial methodological heterogeneity in data processing, network estimation, and interpretation. Most available studies relied on cross-sectional designs, used FFQs as primary assessment tools, and varied widely in sample characteristics and health outcomes examined. The authors emphasized several challenges, including inconsistent reporting of analytical parameters, limitations of centrality metrics for dietary networks, and inadequate handling of the non-normal and zero-inflated nature of dietary data. The authors concluded about the potential of network analysis to capture the complexity of dietary behaviors and the need for standardized practices to advance its application in nutritional science.

The network obtained in this study had a density of 0.368, suggesting moderate connectivity between nodes. Although not all nodes are directly connected, as weak relationships were not modeled, the amount of moderate to strong relationships was relatively high. The diameter of the network was 3, which means that a maximum of three nodes need to be traversed to get from any one to another in the network. This suggests a relatively compact and efficient connection between variables in this network.

Regarding modularity, the clustering global index was 0.40, which is within the acceptable range, indicating that the network is divided into three well-defined groups, or modules. These communities grouped related nodes, so functional patterns or subnetworks could be identified within the global structure. The first group contains variables related to insulin, glucose and the HOMA-IR index, reflecting a set of metabolic variables. The second group includes cholesterol and triacylglycerol, which are key indicators of lipid profile [73]. The third group includes the remaining network variables, suggesting a structure that groups sociodemographic, psychological and nutritional variables into a single module. This underscores the importance of how the different (metabolic, psychological and sociodemographic) dimensions of health are interrelated. Indeed, previous studies support our findings, indicating that metabolic disorders, such as insulin resistance, dyslipidemia and HTN, are common and are exacerbated by inflammation and endocrine disruption associated with excess body fat [74,75]. In turn, psychological factors, such as stress, anxiety and depression, influence dietary habits and may increase metabolic risk through mechanisms such as increased cortisol [76,77,78]. Meanwhile, sociodemographic variables, such as sex, age, marital status and socioeconomic level, impact the distribution of body fat, access to healthy foods and exercise opportunities, contributing to obesity [79,80,81,82]. As these dimensions are interrelated, we can conclude that metabolic factors do indeed aggravate psychological and social difficulties, and vice versa, creating a vicious circle that makes obesity more difficult to control.

From our analysis of centrality indices, we were able to identify the most influential nodes in the network. Stress had the highest centrality in all three measures (eigenvector, interrelatedness, and closeness), highlighting its crucial role as a bridge node connecting several variables to each other. Indeed, activation of this node influences multiple variables, including anxiety, NP (NP1 and NP3), HTN, and several metabolic indicators, including glucose and cholesterol. This finding is consistent with previous studies showing that stress can exacerbate metabolic and psychological health issues, and underscores the need for overweight and obese individuals to manage their stress [83,84]. Previous studies confirm our findings by linking metabolic stress to increased insulin resistance, appetite disorders, lipid profile modification and inflammation, all of which thus raise the risk of obesity, diabetes and cardiovascular diseases [84]. Psychological studies also support our findings by indicating that stress is linked to anxiety disorders, depression, mental fatigue, sleep disorders and destructive behaviors such as substance abuse [85]. These effects are mutually reinforcing, affecting both physical and emotional well-being. Importantly, the activation of nodes in our study should be interpreted as reflecting associations rather than directional or causal effects, given the cross-sectional nature of the design.

The node with the second highest centrality was NP1 (vitamins and minerals), which also had a strong connection with other variables in the network, particularly BMI, glucose, and high blood pressure. Activation of this node significantly influenced other metabolic and psychological variables, indicating the importance of essential nutrients for regulating metabolism and its relationship with mental health [86,87]. Again, recent studies support our findings on the crucial role of vitamins, minerals, essential fatty acids, and amino acids in regulating metabolism, since they are essential for processes such as energy production, protein synthesis, and enzymatic function [88]. Furthermore, these nutrients have a close relationship with mental health, as they influence the production of key neurotransmitters such as serotonin, dopamine, and GABA, which regulate mood, stress, and cognition [89]. Deficiencies in these nutrients are thought to disrupt metabolism, contribute to metabolic disorders, and increase the risk of anxiety, depression, and other psychological problems [90].

Finally, HTN is another high-centrality node, especially in terms of interrelationships, and hence plays a pivotal role in the network. HTN directly affected several NPs, such as NP1 (vitamins and minerals) and NP2 (carbohydrates), as well as other metabolic indicators, such as BMI and triacylglycerols. This underscores the relevance of arterial hypertension as an important marker of metabolic health and its interaction with other risk factors [91,92]. Its presence not only reflects imbalanced blood pressure regulation but is also closely associated with several metabolic risk factors, such as obesity, insulin resistance, dyslipidemia, and chronic inflammation. Consistently high blood pressure can damage blood vessels, increase heart strain, and potentially lead to heart attacks, strokes, and kidney failure. Hypertension also interacts in a complex manner with other risk factors. Obesity, for example, raises blood pressure by increasing vascular resistance, while insulin resistance and high lipid levels (such as triglycerides and LDL cholesterol) promote atherosclerosis, a process that narrows and hardens the arteries, worsening hypertension. So, hypertension is not only a risk factor in itself but is also a crucial marker of a broader metabolic syndrome that significantly increases the risk of chronic diseases [93,94,95].

### 4.1. Strengths

A key novelty of the present study lies in the application of network analysis to the field of nutrition, where the use of this methodology remains relatively limited compared to its widespread adoption in psychiatry and broader medical research. Previous research in nutrition and obesity has predominantly relied on traditional approaches that consider variables in isolation or focus on unidimensional assessments. In contrast, our study integrates multiple domains of information (including anthropometric, behavioral, metabolic, and psychological measures) within a single network framework. This multidimensional approach allows for the examination of complex interrelations and potential causal pathways among factors contributing to weight status and health-related behaviors. Importantly, the analysis is conducted in a high-risk population of young university students with overweight or obesity, a demographic in which early detection of interconnected behavioral and physiological mechanisms may inform targeted prevention and intervention strategies. By combining multidimensional data with network modeling, this study provides a comprehensive and mechanistically informative perspective on the systemic interactions underlying obesity-related outcomes in young adults.

### 4.2. Limitations and Proposals for Future Research

Although our results are revealing, the study does have certain limitations. First, network analysis in this study cannot capture the full complexity of causal relationships between variables: given the cross-sectional design, associations between nodes do not provide information on directionality; thus, the observed connections should be interpreted as statistical correlations rather than indicative of causal effects. Future research employing longitudinal or experimental designs is needed to explore how these relationships evolve over time. Also, our analysis was based on a sample of university students, which limits the generalizability of the findings to other demographic groups. In particular, the present study cannot determine how quickly mental stress might begin to affect physiological or metabolic variables, highlighting the need for longitudinal investigations to capture the onset and temporal dynamics of these effects. A further limitation of the study is the reliance on self-reported dietary data, which may be subject to recall bias, misreporting, and inaccuracies in estimating portion sizes (although validated instruments were used to reduce such errors, the potential for measurement bias should be considered when interpreting associations between dietary intake and cardiometabolic outcomes).

Future research could expand this analysis by including additional variables, such as level of physical activity, to obtain a more complete view of the factors that influence the health of overweight and obese individuals. It would also be useful to explore how interventions aimed at modifying one or more of the central variables (such as stress or high blood pressure) might affect the structure of the network and long-term health outcomes.

## 5. Conclusions

This network analysis provides a comprehensive view of the complex interrelations among nutritional, metabolic, psychological, and sociodemographic factors in overweight and obese university students. The findings highlight the central role of stress, nutritional patterns (NP), and high blood pressure, underscoring the need for integrated strategies that address the interactions among these factors to promote health and prevent diseases associated with overweight and obesity.

Specifically, the results suggest that stress reduction may have beneficial effects not only on mental health but also on metabolic and nutritional variables in overweight and obese young adults. Given the central position of stress within the network, interventions targeting this factor could lead to meaningful improvements in overall health. In addition, the relevance of nutritional patterns (particularly NP1, related to vitamins and minerals) indicates that diet quality plays a crucial role in metabolic health and in reducing obesity-related risks. Therefore, interventions aimed at increasing the intake of essential micronutrients should be considered a key component of prevention and treatment strategies. Finally, arterial hypertension emerged as a central variable influencing multiple health domains, suggesting that its management through dietary modification, increased physical activity, and stress management may have a broad positive impact on the health of overweight university students.

The results also underscore the importance of implementing early detection, assessment, and intervention programs for psychological distress within university settings, especially among students in the early stages of their academic trajectory, as well as the development of targeted prevention programs for subgroups at higher vulnerability.

## Figures and Tables

**Table 1:** Results of the network.

ID	Closeness Centrality	Harmonic Closeness Centrality	Betweenness Centrality	Authority	HUB	Modularity	Clustering. Coefficient	Number Triangles	Eigenvector Centrality
Sex	0.5926	0.6979	6.6262	0.2829	0.2829	3	0.3810	8	0.7852
Age	0.5000	0.5833	1.5056	0.1632	0.1632	3	0.5000	3	0.4526
Marital	0.5333	0.6458	4.4500	0.2141	0.2141	3	0.4000	6	0.5945
Social	0.5517	0.6354	2.7444	0.2161	0.2161	3	0.3000	3	0.6008
BMI	0.5926	0.6771	8.1968	0.2434	0.2434	3	0.2667	4	0.6811
HTN	0.6667	0.7500	12.8881	0.3399	0.3399	3	0.3929	11	0.9491
Glucose	0.5926	0.6771	7.3889	0.2336	0.2336	2	0.4667	7	0.6604
Insulin	0.4571	0.5521	0.3333	0.1237	0.1237	2	0.8333	5	0.3578
CholesT	0.6154	0.7083	8.8746	0.2521	0.2521	1	0.4762	10	0.7153
TAG	0.4848	0.5729	1.3111	0.1382	0.1382	1	0.6667	4	0.3961
HOMA	0.5333	0.6250	5.2429	0.1606	0.1606	2	0.5000	5	0.4608
Depre	0.5000	0.5833	1.6500	0.1587	0.1587	3	0.3333	2	0.4406
Anxiety	0.5926	0.6771	4.2667	0.2658	0.2658	3	0.4667	7	0.7377
Stress	0.6957	0.7813	17.3468	0.3589	0.3589	3	0.3333	12	1.0000
NP1	0.6667	0.7500	7.9389	0.3429	0.3429	3	0.4286	12	0.9536
NP2	0.6400	0.7188	12.8690	0.2710	0.2710	3	0.2857	6	0.7552
NP3	0.5161	0.5938	1.3667	0.1792	0.1792	3	0.5000	3	0.4965

Note. Nodes are identified as: Sex (biological sex), Age (chronological age), Marital (marital status), Social (social position index), NP1 (nutritional pattern 1, minerals and vitamins), NP2 (nutritional pattern 2, carbohydrates), NP3 (nutritional pattern 3, fat and sodium), BMI (body mass index), HTN (hypertension), Glucose, Insulin, CholesT (cholesterol total), TAG (triacylglycerol), HOMA (HOMA-IR index), Depre (depression), Anxiety, Stress.

## Data Availability

The raw data supporting the conclusions of this article will be made available by the authors, without undue reservation.
